# Approach to Patients with Obesity and Other Cardiovascular Risk Factors in Primary Care Using the Delphi Methodology

**DOI:** 10.3390/jcm11144130

**Published:** 2022-07-16

**Authors:** Pedro Morillas Blasco, Silvia Gómez Moreno, Tomás Febles Palenzuela, Vicente Pallarés Carratalá

**Affiliations:** 1 Cardiology Service, Hospital General Universitario de Elche, 03203 Alicante, Spain; pedromorillas68@gmail.com; 2 Cardiology Service, Virgen del Rocío University Hospital, 41013 Seville, Spain; silviagomezmoreno@gmail.com; 3 Department of Medicine, University of Seville, 41004 Sevilla, Spain; 4 Intensive Care Unit, Hospiten Sur, 38001 Santa Cruz de Tenerife, Spain; tomas.febles@hospiten.com; 5 Health Surveillance Unit, Castellon Mutual Insurance Union, 12004 Castellón, Spain; 6 Department of Medicine, Jaume I University, 12071 Castellón, Spain

**Keywords:** cardiovascular risk factors, Delphi method, ischemic heart disease, obesity

## Abstract

Background: Implementing preventive strategies for patients with obesity would improve the future burden of cardiovascular diseases. The objective was to present the opinions of experts on the approach to treating patients with obesity and other cardiovascular risk factors from a primary care perspective in Spain; Methods: Using the Delphi technique, a 42-question questionnaire was developed based on results from the scientific literature, and sent to 42 experts in primary care. Two rounds of participation were held; Results: There is a close relationship between obesity and cardiovascular risk factors among primary care physicians. It is necessary to use a checklist in primary care that includes metabolic parameters such as body mass index, waist circumference, and levels of C-reactive protein and ferritin. It is also useful to combine pharmacological treatment, such as liraglutide, with a change in lifestyle to achieve therapeutic goals in this population; Conclusions: There is a high level of awareness among experts in Spain regarding obesity and other cardiovascular risk factors, and the need to address this pathology comprehensively. The need to incorporate specific tools in primary care consultations that allow for better assessment and follow-up of these patients, such as cuffs adapted to arm size or imaging techniques to assess body fat, is evident. Teleconsultation is imposed as a helpful tool for follow-up. Experts recommend that patients with obesity and associated comorbidities modify their lifestyle, incorporate a Mediterranean diet, and administer liraglutide.

## 1. Introduction

Obesity is considered a multifactorial disease in which environmental and genetic factors interact [1]. Increasingly sedentary lifestyles [2] and unhealthy diets [3] mean that obesity is increasing in prevalence in developed countries, and is considered a serious public health problem [4].

Although there are various anthropometric measurements [5], in practice, obesity is diagnosed as a body mass index (BMI) ≥ 30 kg/m^2^, although if the BMI is between 25 and 29.9 kg/m^2^ [6], it is recommended to complement the assessment with a waist circumference (WC) measurement, since this allows for the estimation of visceral obesity and cardiometabolic risk [7,8,9].

Obesity is associated with an increased risk of cardiovascular disease (CVD), particularly ischemic heart disease and heart failure, including atrial fibrillation, ventricular arrhythmias, and sudden death [10]. The increased risk of CVD, particularly atherosclerotic CVD, among people with obesity is largely mediated by established traditional risk factors, such as insulin resistance, type 2 diabetes mellitus, dyslipidemia, hypertension, and obstructive sleep apnea [11]. In addition, obesity can be considered a low-grade chronic inflammatory pathology, where visceral and epicardial adipose tissue generate high plasma levels of proinflammatory cytokines such as tumor necrosis factor-alpha (TNF-α) or C-reactive protein [12].

CVDs are the leading cause of morbidity and mortality worldwide, which is why they are a cause for particular concern, since they also place significant pressure on healthcare systems and cause a loss of work productivity and poor quality of life for the patient [13]. The timely implementation of preventive strategies for patients with obesity would improve the future burden of CVD related to weight and the burden of medical care [14].

Different studies have evaluated other therapeutic options in patients with obesity, such as lifestyle [15,16,17], pharmacological treatment [18], and surgical treatment in cases of significant obesity [19,20], all with disparate results.

Other consensus studies have evaluated approaches to reduce cardiovascular risks, such as in patients with type 2 diabetes [21], the use of the polypill in cardiovascular disease [22], or the contributors to cardiometabolic disease [23]; however, similar studies have not been conducted among participants with obesity.

This study aimed to reach a consensus on the approach to patients with obesity and other cardiovascular risk factors from a primary care (PC) perspective in Spain.

## 2. Materials and Methods

### 2.1. Study Design

A survey of experts was conducted using the Delphi methodology. This technique is a structured process that uses a series of questionnaires sent to a set of experts in at least two rounds to collect information [24]. This methodology is one of the most commonly used to evaluate the collective and anonymous opinion of the members of a panel of experts to examine the evidence and to reach a consensus on issues that did not previously exist [25,26].

A review of the scientific literature was conducted to identify evidence gaps supporting the content of the survey. The steering committee directed the development of the surveys for each round of voting, reviewed the responses and summaries collected, validated the systematic literature search, and critically appraised the evidence. A total of 42 initial questions were asked, which were distributed in the following blocks of knowledge: (1) Evaluation of the degree of incidence of obesity and associated cardiovascular risk factors, (2) evaluation of barriers in the diagnosis, prescription, and follow-up of these patients by the primary care physician or specialist, (3) improvement in obesity-related parameters in a patient being treated with lipid-lowering and antihypertensive drugs, and (4) analysis of improvements in cardiovascular parameters in responding patients under pharmacological treatment and/or bariatric surgery treatment.

### 2.2. Participants

The research team consisted of three cardiologists who work in different areas (clinical, intensive, and cardiac rehabilitation) and a family doctor with experience in managing patients with high or very high cardiovascular risk.

The members of the panel of experts were chosen based on having at least 20 years of experience in the care of patients with obesity and cardiovascular risk factors, who worked in primary care centers, and two or three doctors were chosen, representing the different Autonomous Communities of Spain. The expert panel initially consisted of 42 primary care experts. Participants were invited via email. Information about the study and a link to the survey Case Report Form (CRF) were provided.

**Figure 1:**
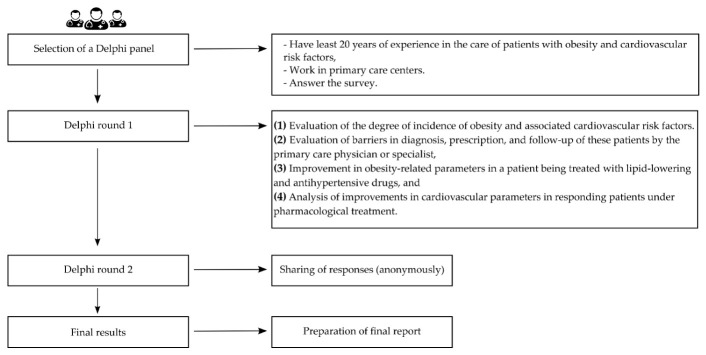
Delphi design study flow chart.

The responses provided in the Round 1 survey were shared in Round 2 as a collective list. This information was shared with the rest of the participants anonymously. Two rounds were conducted for the experts: the first between 15 October and 18 November 2021, and the second between 14 December and 17 January 2022. Figure 1 shows the steps of the study design.

### 2.3. Validation and Ethical Aspects

Multiple principal investigators and family physicians validated the survey questions in the near-work setting for their input. All procedures were performed following the relevant guidelines of the Declaration of Helsinki, and were approved by the Review Committee of the Spanish Society of Primary Care Physicians (SEMERGEN).

### 2.4. Statistical Analysis

Questions with discrete quantitative answers for each item were evaluated using a Likert scale from 0 to 10 points (0 = completely disagree; 10 = completely agree). The consensus criterion used was as follows: for agreement, a median ≥ 8 and an interquartile range (IQR) < 0.4; for disagreement, a median ≤ two and an IQR < 0.4.

The questions with categorical answers were evaluated through the distribution of frequencies and percentages. The consensus criterion for agreement for questions with nominal categorical answers was that one of the answers accounted for at least 50% of the total responses, and for questions with a dichotomous categorical answer or with several categories with multiple answers, one of the responses accounted for at least 70% of the total responses.

Data were analyzed using Gandia Barbwin version 7.0.2110.5 (Tesi S.L., Gandia, Valencia, Spain) and XLSTAT^®^ version 21.04 (Addinsoft SARL, Paris, France) of Microsoft Excel^®^.

## 3. Results

**Figure 2:**
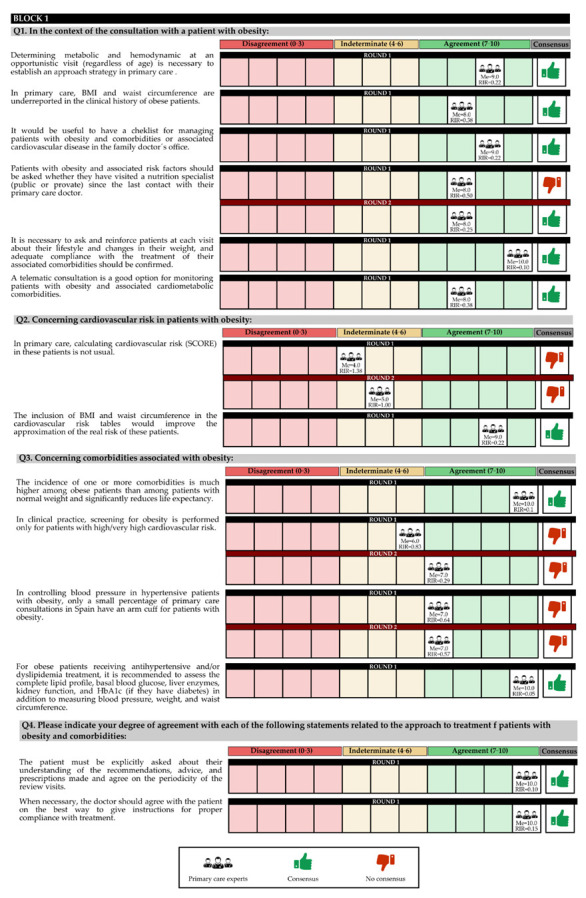
Discrete quantitative response questions of Block 1 under the Likert scale. IQR: Interquartile range; Me: Median.

**Figure 3:**
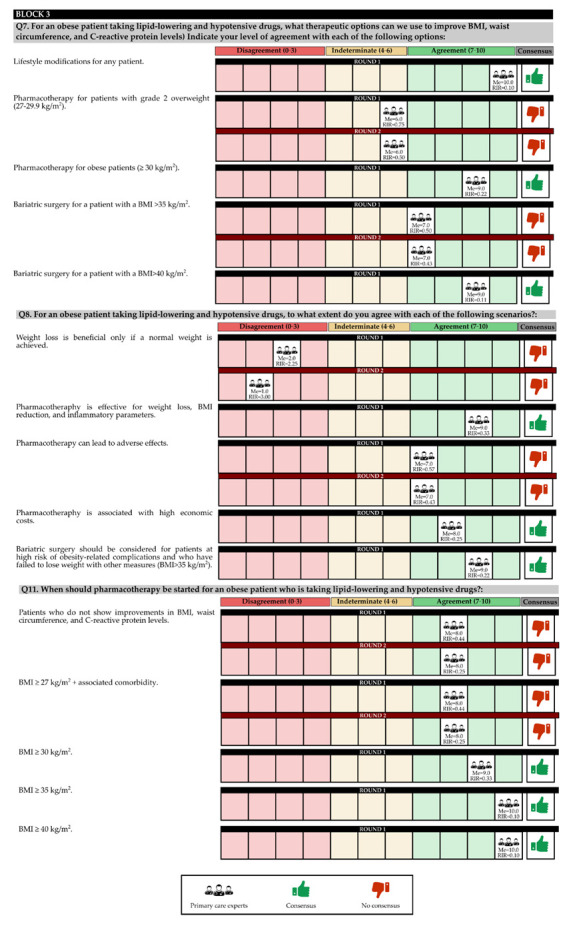
Discrete quantitative response questions of Block 2 under the Likert scale. IQR: Interquartile range; Me: Median.

**Figure 4:**
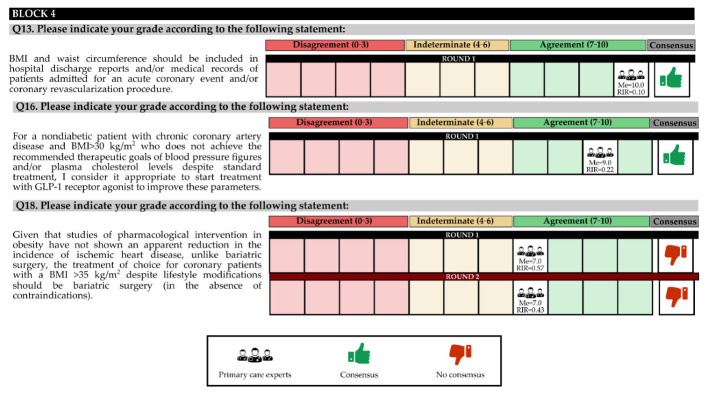
Discrete quantitative response questions of Block 3 under the Likert scale. IQR: Interquartile range; Me: Median.

In the first round, 97.62% of the experts contacted participated, and in the second round, 73.81% participated. The questions and answers are shown in Figure 2, Figure 3 and Figure 4 and Table 1 and Table 2.

### 3.1. BLOCK I. Evaluation of the Degree of Incidence of Obesity and Associated Cardiovascular Risk Factors

The experts concluded that for an obese patient, regardless of age, it is necessary to assess metabolic and hemodynamic parameters in an opportunistic visit. It was recommended to establish an approach strategy in PC through a checklist. The experts indicated that BMI and WC are underreported, and that it would be desirable to include them in cardiovascular risk tables to better calculate the probability of risk.

On the other hand, the patient must be asked and reinforced at each visit regarding their lifestyle and changes in their weight, and adequate compliance with the treatment must be confirmed, with telematic consultation being a good option for follow-up. Additionally, it is necessary to ensure that the patient understands the information provided by the doctor in order to perform the treatment correctly, and agrees on the periodicity of the review visits.

Experts consider that the incidence of one or more comorbidities is much higher among obese patients than among patients of normal weight, and significantly reduces life expectancy. On the other hand, for obese patients receiving antihypertensive and/or dyslipidemia treatment, it is recommended to evaluate the complete lipid profile, basal glycemia, liver enzymes, renal function, and HbA1c (if they have diabetes), in addition to measuring blood pressure, weight, and WC.

Finally, there was consensus in the second round that patients with obesity and associated risk factors should be asked whether they have visited a nutrition specialist (public or private) since the last contact with their PC doctor.

On the other hand, no consensus was reached on using the cardiovascular risk calculation tool (SCORE). In addition, screening is conducted only for patients with a high/very high cardiovascular risk, and blood pressure measurement is performed only for a small percentage of patients, since an arm cuff for patients with obesity is not available in most outpatient PC clinics (Supplementary Material Table S1).

In the first round, the degree of agreement was 71.43%, and in the second round, there was no final consensus for 21.43% of the total questions in the block.

### 3.2. BLOCK II. Evaluation of Barriers in Diagnosis, Prescription, and Follow-Up by the Primary Care Physician or Specialist

The experts did not reach a consensus on the possible barriers to using liraglutide 3.0 mg in the PC field. In the second round, only on the part of the doctors did the experts reach a consensus of agreement where they identified the frequency of daily administration of the drug as a barrier.

On the patient’s side, in the first round, the experts reached a consensus on the patient’s fear of regaining weight after stopping treatment as a possible barrier to using liraglutide. However, in the second round, the experts did not agree on this item.

In the first round, the degree of agreement was 10.00%, and in the second round, there was no final consensus for 90.00% of the total questions in the block.

### 3.3. BLOCK III. Improvement of Obesity-Related Parameters in a Patient Being Treated with Lipid-Lowering and Antihypertensive Drugs

The experts agreed that for an obese patient who is taking lipid-lowering and hypotensive drugs, there are improvements in BMI, WC, and C-reactive protein; if lifestyle changes occur, pharmacotherapy should be administered even if it has a high economic cost, and bariatric surgery should be performed on patients with a BMI > 40 kg/m^2^ and who have failed to lose weight with other measures.

According to the experience of the experts, pharmacotherapy should be started for patients with a BMI ≥30 kg/m^2^, with the best starting guideline being the administration of liraglutide, accompanied by changes in lifestyle. Additional laboratory parameters that should be measured are C-reactive protein and ferritin levels.

There was no consensus on administering pharmacotherapy to patients with grade 2 overweight (BMI ≥ 27–29.9 kg/m^2^), or on performing bariatric surgery on patients with a BMI of between 35 and 40 kg/m^2^. In addition, there was no consensus among the experts as to the best order of prescription of metformin, orlistat, or liraglutide for improving weight, WC, and C-reactive protein levels. In contrast, there was a consensus that it was not necessary for the patient to reach a normal weight to obtain beneficial results. In addition, they assumed that pharmacotherapy could lead to adverse effects (Supplementary Material Table S2).

In the first round, the degree of agreement was 52.38%, and in the second round, there was no final consensus for 38.10% of the total questions in the block.

### 3.4. BLOCK IV. Analysis of Improvements in Cardiovascular Parameters in Responding Patients under Pharmacological Treatment

The experts agreed that BMI and WC should be included in hospital discharge reports and/or medical records of patients admitted for an acute coronary event and/or coronary revascularization procedure. Likewise, they considered it necessary to start treatment with a glucagon-like peptide-1 (GLP-1) receptor agonist to improve these parameters in nondiabetic patients with chronic coronary disease and a BMI > 30 kg/m^2^, for whom the therapeutic goals of blood pressure and/or plasma cholesterol levels are not recommended, despite standard treatment.

Experts consider that the goal of weight loss in patients with overweight or obesity grade 1 should be approximately 5–10%. On the other hand, for a patient with a BMI > 30 kg/m^2^ who has suffered a coronary event, it is better to initially combine pharmacological treatment with lifestyle changes.

In the second round, the experts agreed that for some selected patients, when proposing a more intensive treatment, it could be helpful to use an imaging technique that provides information on the distribution and characteristics of visceral fat in ischemic patients with obesity (e.g., liver and pericardial ultrasound, axial computed tomography, and magnetic resonance imaging). In addition, they recommended a Mediterranean style diet (enriched with olive oil and nuts) for patients with obesity and coronary heart disease.

There was no consensus that the treatment of choice for coronary patients with a BMI > 35 kg/m^2^, despite lifestyle changes, should be bariatric surgery (in the absence of contraindications) (Supplementary Material Table S3).

In the first round, the degree of agreement was 57.14%, and in the second round, there was no final consensus for 14.29% of the total questions in the block.

## 4. Discussion

This consensus is the first Spanish study published in the medical literature that addresses the management of patients with obesity and other risk factors associated with CVD. In general, the experts reached a consensus on the association between obesity and cardiovascular risk factors, and the clinical parameters that improve in these patients treated with both lipid-lowering and antihypertensive drugs, and with drugs for weight reduction. However, they did not reach an agreement on the best method for assessing cardiovascular risk in this population, the best treatment for patients with grade 2 overweight, or the role of bariatric surgery in patients with ischemic heart disease and a BMI within a range of 35–40 kg/m^2^.

Concerning the diagnosis, it is recommended to assess patients with obesity in PC through the use of a checklist. “Checklists” have been used as a public health strategy [27,28] and would allow an evaluation of the factors involved in CVD development. The scientific literature supports using BMI and WC, although it would also be desirable to add the SCORE risk tables [29,30]. The SCORE tables estimate the risk of cardiovascular mortality in subjects aged 40 to 65. They are simple to use, since they include a few parameters such as age, sex, blood pressure, total cholesterol, high-density lipoprotein cholesterol, and smoking [29]. However, one of the great limitations is that they do not allow for calculating the risk beyond 65 years [31].

In the results of this consensus, the measurement of C-reactive protein and ferritin levels is also recommended when evaluating patients with obesity and assessing whether they have other cardiovascular risk factors. Obesity is characterized by a state of chronic inflammation, and it has been documented that C-reactive protein is strongly associated with the pathology [32]. On the other hand, serum ferritin levels are positively associated with type 2 diabetes mellitus, coronary artery disease, and cerebrovascular disease [33].

Concerning the treatment, the experts consider that a modification in lifestyle and the administration of liraglutide is the best alternative, all accompanied by adequate follow-up. However, there are many international societies that, far from incorporating conventional therapies, support bariatric and metabolic surgeries as the most effective treatments [34]. There is consensus in considering bariatric surgery for those patients with a BMI ≥ 40 kg/m^2^, since it has a beneficial effect on metabolic parameters with a significant reduction in BMI, systolic blood pressure, triglycerides, and fasting glucose levels [35,36].

Unlike other studies, this consensus included other comorbidities such as hypertension, high cholesterol, or diabetes. Eleazu C, et al. [37] suggest that treatments should include the monitoring of associated comorbidities to assess the therapeutic success of the obese patient.

Among the important points addressed, the great importance of continuous monitoring of treatment in obese patients is identified. In this sense, telematic consultation becomes a work tool that has been reinforced during the COVID-19 pandemic [38,39].

Secondly, the experts indicate that one of the main barriers to the adequate clinical assessment of obese patients is the lack of arm cuffs adapted to the size of the patient’s arm, limiting adequate control and follow-up, especially in patients with arterial hypertension. Several guidelines report obesity as a risk factor that influences the development of arterial hypertension [40,41,42]. Family physicians should worry about having adequate cuffs for patients with obesity, and demand that their managers provide adequate material for managing these patients.

Third, the experts emphasize the importance of having specific imaging techniques, such as having ultrasound available during the consultation to assess the body and visceral fat, as they can be used to quantify the distribution of adipose tissue [43,44,45]. It would also be beneficial to have an ultrasound for the identification, for example, of hepatic steatosis, including this examination, in the evaluation of patients with suspected cardiometabolic risk. All of these tools would aim to reduce the progression of the disease and implement appropriate measures such as changes in lifestyle.

Fourth, although the pharmacological treatments considered were metformin, orlistat, liraglutide, and the combination of orlistat together with liraglutide, the experts consider lifestyle modification and the administration of liraglutide to be the best alternative. Up to 73.2% of the experts agree that the best treatment starting guideline to reduce BMI, WC, and C-reactive protein levels for an obese patient taking lipid-lowering and hypotensive drugs is the administration of liraglutide, accompanied by changes in lifestyle. The experts recommended adopting the Mediterranean diet and supplementing in fats with the addition of extra virgin olive oil or nuts [46]. Other studies have shown that this reduces cardiovascular and cerebrovascular morbidity and mortality. In addition, combining the Mediterranean diet and exercise improves overweight/obesity and metabolic syndrome [47]. Concerning liraglutide, it is an agonist of the human GLP-1 receptor, which plays an essential role in the resistance to obesity [48,49]. Numerous clinical trials have observed that the administration of liraglutide at 3.0 mg per day to obese patients and most importantly, accompanied by a change in lifestyle, significantly reduces visceral adipose tissue over 40 weeks of treatment [50,51,52].This study was conducted following the Delphi methodology, which inherently presents limitations in validity and reliability. It is a very laborious process that requires at least two rounds to obtain an adequate consensus, subjective criteria are developed that are subject to external influences from the participants, and there may be confusion in the interpretation of the content of some questions. However, this methodology has become an essential part of addressing problems and making decisions in health services [53].

Lastly, the administration of liraglutide, despite its price and the lack of financing from the Public Health System, became the best option after the different studies were conducted. However, there is still a lack of agreement on bariatric surgery in BMI > 35–39.9 kg/m^2^, possibly due to significant variability between the different autonomous communities and health areas in Spain.

## 5. Conclusions

There is a high level of awareness among experts in Spain regarding obesity and other cardiovascular risk factors, and the need to address this pathology comprehensively. The need to incorporate specific tools in PC consultations that allow for better assessment and follow-up of these patients, such as arm cuffs adapted to their size, or imaging techniques to assess body fat, is evident. Teleconsultation is imposed as a helpful tool for follow-up. Lastly, experts recommend that patients with obesity and associated comorbidities modify their lifestyle, incorporate a Mediterranean diet, and administer liraglutide.

## Figures and Tables

**Table 1:** Response-ordering questions. CV: Coefficient of variation.

**BLOCK 2**	**FIRST ROUND**			**SECOND ROUND**		
	**Mean**	**CV**	**Consensus**	**Mean**	**CV**	**Consensus**
**Q5. Please indicate the relevance of the potential barriers to the use of liraglutide 3.0 mg BY THE PRIMARY CARE PHYSICIAN:**						
The low perception of obesity as an important cardiometabolic risk factor in primary care.	2.90	0.50	No	2.70	0.52	No
The lack of financing of the drug by Social Security.	4.10	0.38	No	4.00	0.40	No
The need for patient control visits at the beginning of treatment to monitor weight loss and adjust the dose.	2.80	0.50	No	2.60	0.54	No
Subcutaneous administration of the drug.	2.60	0.36	No	2.90	0.42	No
The frequency of daily administration of the drug.	2.60	0.45	No	2.80	0.30	Yes
**Q6. Please indicate the relevance of the potential barriers to the use of liraglutide 3.0 mg BY PRIMARY CARE PATIENTS:**						
Rejection of pharmacological treatment for obesity by the patient.	2.20	0.67	No	2.20	0.64	No
The patient’s fear of regaining weight when stopping treatment.	3.10	0.30	Yes	2.80	0.34	No
The patient fears that they may abandon the treatment or that it may become an indefinite treatment.	3.00	0.35	No	2.80	0.41	No
Subcutaneous administration of the drug.	2.60	0.48	No	3.20	0.37	No
The price of the treatment.	4.1	0.39	No	4.1	0.41	No
**BLOCK 3**	**FIRST ROUND**			**SECOND ROUND**		
	**Mean**	**CV**	**Consensus**	**Mean**	**CV**	**Consensus**
**P9. For an obese patient who is taking lipid-lowering and hypotensive drugs, to what extent do you think it is appropriate to use each of the following pharmacological options to improve BMI parameters, waist circumference, and C-reactive protein levels?:**						
Metformin.	2.20	0.58	No	1.90	0.58	No
Orlistat.	2.30	0.34	No	2.60	0.34	No
Liraglutide.	3.00	0.38	No	3.00	0.46	No
Orlistat + liraglutide.	2.50	0.45	No	2.60	0.31	No

**Table 2:** Categorical response questions.

**BLOCK 3**	**FIRST ROUND**	**SECOND ROUND**
**Q10. Based on experience, what would be the best starting treatment guideline for reducing BMI parameters, waist circumference, and C-reactive protein levels for an obese patient taking lipid-lowering and hypotensive drugs?:**	**%**	**%**
Liraglutide + lifestyle changes.	73.2	
Orlistat + lifestyle changes.	4.9	
Metformin + lifestyle changes.	7.3	
Liraglutide + orlistat + lifestyle changes.	14.6	
**Q12. What additional laboratory parameters do you think should be measured in obese patients who are taking lipid-lowering and hypotensive drugs?:**	**%**	**%**
C-reactive protein.	97.6	
Ferritin.	70.7	
Fasting insulin.	61.0	
Homocysteine.	34.1	
Fibrinogen.	26.8	
**BLOCK 4**	**FIRST ROUND**	**SECOND ROUND**
**Q14. Since visceral fat is a prothrombotic and proinflammatory risk marker, should an imaging technique be incorporated into routine practice to obtain information on the distribution and characteristics of visceral fat in obese ischemic patients (e.g., hepatic ultrasound, pericardial ultrasound, axial computed tomography** **, or magnetic resonance imaging)?:**	**%**	**%**
No, it does not provide relevant information for the management and follow-up of these patients.	9.8	3.2
It could be useful to propose a more intensive treatment for some selected patients.	48.8	67.7
Yes, because it provides relevant information that can influence these patients’ prognosis and/or treatment.	41.5	29.0
**Q15. To achieve a direct impact on survival in the medium–long term, and given the absence of clinical trials specifically focused on it, what should be the weight loss goal for patients with grade 1 overweight or obesity (BMI < 35 kg/m^2^) and chronic ischemic heart disease?**	**%**	**%**
No goal. Several studies have shown that subjects with established coronary disease and grade 1 overweight or obesity have a better prognosis than subjects with normal or low weight (obesity paradox).	0	
Weight reduction < 5%.	2.4	
5–10% weight reduction.	53.7	
Weight reduction ≥ 10%.	43.9	
**Q17. For a patient with a BMI > 30 kg/m^2^ who has suffered a coronary event, should we initially propose a specific pharmacological treatment associated with lifestyle changes, or is a more staggered approach preferable, such as introducing drugs later if weight goals are not achieved?:**	**%**	**%**
Due to the potential negative prognostic impact of obesity in this high-risk patient, it is better to combine pharmacological treatment with lifestyle modification initially.	61.0	
Stepwise management is preferable: start lifestyle modifications (diet + physical exercise + behavior modification) and introduce drugs at 3–6 months if the objectives are not achieved.	39.0	
**Q19. What do you think should be the recommended diet for coronary patients with obesity?:**	**%**	**%**
Hypocaloric diet.	29.3	16.1
Mediterranean diet enriched with olive oil and nuts.	46.3	74.2
Low-carbohydrate diet.	4.9	3.2
Low-fat diet.	19.5	6.5

## Data Availability

Not applicable.

## Supplementary Materials

The following supporting information can be downloaded at: https://www.mdpi.com/article/10.3390/jcm11144130/s1, Table S1: Discrete quantitative response questions under the Likert scale where there was no consensus; Table S2: Response-ordering questions where there was no consensus; Table S3: Categorical response questions where there was no consensus.
