# Supplementary material for: Approach to Patients with Obesity and Other Cardiovascular Risk Factors in Primary Care Using the Delphi Methodology
Source: J Clin Med. 2022 Jul 16;11(14):4130. doi: 10.3390/jcm11144130 (PMC9324671; doi:10.3390/jcm11144130)
Supplement: Supplementary file 1 [file jcm-11-04130-s001.zip › jcm-1767562-supplementary.pdf]

Journal of Clinical Medicine

Table S1: Discrete quantitative response questions under the Likert scale where there was no consensus.

BLOCK 1	FIRST ROUND			SECOND ROUND		
**Q1. In the context of the consultation with a patient with obesity:**	**Me**	**IQR**	**Consensus**	**Me**	**IQR**	**Consensus**
Patients with obesity and associated risk factors should be asked whether they have visited a nutrition specialist (public or private) since the last contact with their primary care doctor.	8	0.50	No	8	0.25	Yes
**Q2. Concerning cardiovascular risk in patients with obesity:**						
In primary care, calculating cardiovascular risk (SCORE) in these patients is not usual.	4	1.38	No	5	1.00	No
**Q3. Concerning comorbidities associated with obesity:**						
In clinical practice, screening for obesity is performed only for patients with high/very high cardiovascular risk.	6	0.83	No	7	0.29	No
In controlling blood pressure in hypertensive patients with obesity, only a small percentage of primary care consultations in Spain have an arm cuff for patients with obesity.	7	0.64	No	7	0.57	No
**BLOCK 3**	**FIRST ROUND**			**SECOND ROUND**		
	**Me**	**IQR**	**Consensus**	**Me**	**IQR**	**Consensus**
**Q7. For an obese patient taking lipid-lowering and hypotensive drugs, what therapeutic options can we use to improve BMI, waist circumference, and C-**						

**reactive protein levels? Indicate your level of agreement with each of the following options:**						
Pharmacotherapy for patients with grade 2 overweight (27-29.9 kg/m^2).	6	0.75	No	6	0.50	No
Bariatric surgery for a patient with a BMI > 35 kg/m^2.	7	0.50	No	7	0.43	No
**Q8. For an obese patient taking lipid-lowering and hypotensive drugs, to what extent do you agree with each of the following scenarios?:**						
Weight loss is beneficial only if a normal weight is achieved.	2	2.25	No	1	3.00	No
Pharmacotherapy can lead to adverse effects.	7	0.57	No	7	0.43	No
**Q11. When should pharmacotherapy be started for an obese patient who is taking lipid-lowering and hypotensive drugs?:**						
Patients who do not show improvements in BMI, waist circumference, and C-reactive protein levels.	8	0.44	No	8	0.25	Yes
BMI ≥27 kg/m^2 + associated comorbidity.	8	0.50	No	8	0.25	Yes
**BLOCK 4**	**FIRST ROUND**			**SECOND ROUND**		
	**Me**	**IQR**	**Consensus**	**Me**	**IQR**	**Consensus**
**Q18. Please indicate your grade according to the following statement:**						

Given that studies of pharmacological intervention in obesity have not shown an apparent reduction in the incidence of ischemic heart disease, unlike bariatric surgery, the treatment of choice for coronary patients with a BMI > 35 kg/m2 despite lifestyle modifications should be bariatric surgery (in the absence of contraindications).	7	0.57	No	7	0.43	No

Table S2: Response-ordering questions where there was no consensus.

BLOCK 2	FIRST ROUND			SECOND ROUND		
	Mean	CV	Consensus	Mean	CV	Consensus
**Q5. Please indicate the relevance of the potential barriers to the use of liraglutide 3.0 mg BY THE PRIMARY CARE PHYSICIAN:**						
The low perception of obesity as an important cardiometabolic risk factor in primary care.	2.90	0.50	No	2.70	0.52	No
The lack of financing of the drug by Social Security.	4.10	0.38	No	4.00	0.40	No
The need for patient control visits at the beginning of treatment to monitor weight loss and adjust the dose.	2.80	0.50	No	2.60	0.54	No
Subcutaneous administration of the drug.	2.60	0.36	No	2.90	0.42	No
The frequency of daily administration of the drug.	2.60	0.45	No	2.80	0.30	Yes
**Q6. Please indicate the relevance of the potential barriers to the use of liraglutide 3.0 mg BY PRIMARY CARE PATIENTS:**						
Rejection of pharmacological treatment for obesity by the patient.	2.20	0.67	No	2.20	0.64	No
The patient's fear of regaining weight when stopping treatment.	3.10	0.30	Yes	2.80	0.34	No
The patient fears that they may abandon the treatment or that it may	3.00	0.35	No	2.80	0.41	No

become an indefinite treatment.						
Subcutaneous administration of the drug.	2.60	0.48	No	3.20	0.37	No
The price of the treatment.	4.1	0.39	No	4.1	0.41	No
**BLOCK 3**	**FIRST ROUND**			**SECOND ROUND**		
	**Mean**	**CV**	**Consensus**	**Mean**	**CV**	**Consensus**
**P9. For an obese patient who is taking lipid-lowering and hypotensive drugs, to what extent do you think it is appropriate to use each of the following pharmacological options to improve BMI parameters, waist circumference and C-reactive protein levels?:**						
Metformin.	2.20	0.58	No	1.90	0.58	No
Orlistat.	2.30	0.34	No	2.60	0.34	No
Liraglutide.	3.00	0.38	No	3.00	0.46	No
Orlistat + liraglutide.	2.50	0.45	No	2.60	0.31	No

Table S3: Categorical response questions where there was no consensus.

BLOCK 3	FIRST ROUND	SECOND ROUND
**Q10. Based on his experience, what would be the best starting treatment guideline for reducing BMI parameters, waist circumference and C-reactive protein levels for an obese patient taking lipid-lowering and hypotensive drugs?:**	**%**	**%**
Orlistat + lifestyle changes.	4.9	
Metformin + lifestyle changes.	7.3	
Liraglutide + orlistat + lifestyle changes.	14.6	
**Q12. What additional laboratory parameters do you think should be measured in obese patients who are taking lipid-lowering and hypotensive drugs?:**	**%**	**%**
Fasting insulin.	61.0	
Homocysteine.	34.1	
Fibrinogen.	26.8	
**BLOCK 4**	**FIRST ROUND**	**SECOND ROUND**
**Q14. Since visceral fat is a prothrombotic and proinflammatory risk marker, should an imaging technique be incorporated into routine practice to obtain information on the distribution and characteristics of visceral fat in obese ischemic patients (e.g., hepatic ultrasound, pericardial ultrasound, axial computed tomography, magnetic resonance imaging)?:**	**%**	**%**
No, it does not provide relevant information for the management and follow-up of these patients.	9.8	3.2
It could be useful to propose a more intensive treatment for some selected patients.	48.8	67.7
Yes, because it provides relevant information that can influence these patients' prognosis and/or treatment.	41.5	29.0
**Q15. To achieve a direct impact on survival in the medium-long term and given the absence of clinical trials specifically focused on it, what should be the weight loss goal for patients with grade 1 overweight or obesity (BMI <35 kg/m^2) and chronic ischemic heart disease?**	**%**	**%**
No goal. Several studies have shown that subjects with established coronary disease and grade 1 overweight or obesity have a better prognosis than subjects with normal or low weight (obesity paradox).	0	

Weight reduction <5%.	2.4	
5-10% weight reduction.	53.7	
Weight reduction ≥10%.	43.9	
**Q17. For a patient with a BMI>30 kg/m^2 who has suffered a coronary event, should we initially propose a specific pharmacological treatment associated with lifestyle changes, or is a more staggered approach preferable, such as introducing drugs later if weight goals are not achieved?:**	**%**	**%**
Due to the potential negative prognostic impact of obesity in this high-risk patient, it is better to combine pharmacological treatment with lifestyle modification initially.	61.0	
Stepwise management is preferable: start lifestyle modifications (diet + physical exercise + behavior modification) and introduce drugs at 3-6 months if the objectives are not achieved.	39.0	
**Q19. What do you think should be the recommended diet for coronary patients with obesity?:**	**%**	**%**
Hypocaloric diet.	29.3	16.1
Mediterranean diet enriched with olive oil and nuts.	46.3	74.2
Low-carbohydrate diet.	4.9	3.2
Low-fat diet.	19.5	6.5
